# Oxidative Stress in Primary Bone Tumors: A Comparative Analysis

**DOI:** 10.7759/cureus.25335

**Published:** 2022-05-25

**Authors:** Vasudha Dhupper, Umesh Yadav, Monica Verma, Kiran Dahiya, Aakriti Chahal, Sushil Kumar, Rahul Kumar, Nishan Yadav

**Affiliations:** 1 Biochemistry, Pandit Bhagwat Dayal Sharma Post Graduate Institute of Medical Sciences, Rohtak, IND; 2 Orthopedics, Pandit Bhagwat Dayal Sharma Post Graduate Institute of Medical Sciences, Rohtak, IND; 3 Biochemistry, Pandit Bhagwat Dayal Sharma Post Graduate Institute of Medical Sciences, ROhtak, IND

**Keywords:** sod, mda, antioxidants, bone tumors, oxidative stress

## Abstract

Background

Bone tumors account for 1% of all cancers and have considerable morbidity and mortality. There is a proposed theory of increased oxidative stress characterized by an increased level of reactive oxygen species (ROS) that disrupts the intracellular reduction-oxidation (redox) balance which has been implicated in various diseases including cancer. The aim of the present study was to measure the levels of oxidant stress and antioxidant mechanism in bone tumors (benign as well as malignant).

Methods

The study cohort consisted of 42 subjects: 14 malignant bone tumors, 14 benign bone tumors, and 14 healthy controls. Serum Malondialdehyde (MDA) levels were determined to assess oxidative stress while antioxidant status was evaluated using superoxide dismutase (SOD).

Results

Patients with malignant bone tumors showed a significant increase in plasma MDA levels (p<0.05) while SOD levels were significantly decreased (p<0.05). No significant difference in oxidative damage was noted between both the sarcomas (p>0.05).

Conclusions

In conclusion, an increase in oxidative stress and a decrease in antioxidant status are observed in bone tumors. Further studies on the manipulation of redox balance in patients with bone tumors can act as a useful approach in early diagnosis or designing management strategies for bone tumors.

## Introduction

Primary neoplasms of the skeleton are rare, amounting to only 0.2% of the overall human tumor load. Bone tumors are classified on the basis of cell origin. Among primary bone tumors, osteochondroma, giant cell tumor, osteoid osteoma, and osteoblastoma are a few commonly seen lesions. Malignant bone tumors are less commonly seen than benign lesions. Osteosarcoma is the most common malignant bone tumor followed by Ewing’s sarcoma and Chondrosarcoma. The gold standard test for confirmation of the diagnosis is biopsy [1].

Although the etiology remains unclear, few environmental factors like radiation, viral infection, chemical exposure, and heritable conditions like Li-Fraumeni syndrome and retinoblastoma have been attributed to the development of malignancy. The role of oxidants and the generation of reactive oxygen species (ROS) in cancer development is still a matter of debate. One of the proposed theories states oxidative stress due to an imbalance between oxidant status and antioxidants might be carcinogenic [2].

A mismatch in oxidative stress and antioxidant status decreases the defense mechanism and produces reactive intermediates which alter the membrane structure and cause lipid peroxidation. Superoxide (O2•−) is one such oxidizing agent which can lead to damage to the structure of DNA, lipids, or proteins. Increased levels of superoxide ions, suggestive of oxidative stress, have been found to be involved in the etiology of multiple disorders like cancer, cardiovascular disorders, and neurodegenerative diseases [3]. Malondialdehyde (MDA) is a product of lipid peroxidation causing cell damage and reacts with the free amino groups of proteins and nucleic acids. The main target of MDA remains to be guanine leading to mutations [4]. Therefore, the determination of MDA can be used to estimate the intensity of oxidative stress or damage caused by lipid peroxidation.

To protect from above mentioned reactive oxygen species (ROS), superoxide dismutases (SODs) play an important role as antioxidant enzymes. Superoxide dismutase converts superoxide into oxygen and hydrogen peroxide and thus diminishes the harmful effect of superoxide and other reactive oxygen species. A significant decrease in antioxidant status (as measured by the decrease in the level of SODs) has been found to be involved in cancer of various sites like the lung, colon, and lymphatic system [4]. A balance between oxidative stress and antioxidant status is crucial for healthy living.

The main aim of the study was to determine the role of oxidative stress and antioxidant status in patients with bone tumors (benign as well as malignant) by assessing the levels of MDA and SOD. This would help in establishing the role of oxidative stress in the etiology of bone tumors for diagnostic as well as therapeutic purposes.

## Materials and methods

This study was carried out in a tertiary care center after obtaining approval from the Institutional Ethical Committee. Fourteen patients with benign bone lesions and fourteen patients with malignant bone lesions were included in the present study. A control group of 14 healthy individuals was also included in the study. Written informed consent was obtained from all patients. The control group was age and sex-matched irrespective of age and gender. Besides plain X-ray, computerized tomography (CT) scan, and magnetic resonance imaging (MRI) was performed for all the patients who underwent biopsy for exact histopathological diagnosis as well as staging. Patients with secondary bone malignancy or metastasis were excluded from the study.

A total of 5ml venous sample was taken from patients with biopsy-proven bone tumors. The same amount of blood (5ml) was also collected from the healthy individuals. Serum samples were later analyzed preferably on the same day.

Determination of lipid peroxidation

Serum MDA was analyzed by a colorimetric method which is based on the principle that 2-thiobarbituric acid (TBA) reacts with MDA when heated at acidic pH. The concentration of MDA was determined and the results were expressed as micromoles of MDA per liter of plasma [5,6].

Determination of Superoxide Dismutase levels (SOD)

Serum SOD activity was measured by the enzymatic method on the Randox autoanalyzer. This method uses xanthine and xanthine oxidase to generate superoxide radicals further leading to the formation of red formazan dye. The action of SOD was calculated by the efficacy of inhibition of this reaction. One unit of SOD causes a 50% inhibition of the rate of reduction. SOD levels were expressed as units per milliliter of plasma [7,8].

The data was compiled and analyzed using a statistically suitable method like the Chi-square test. As a standard practice, a p-value of <0.05 was considered statistically significant.

## Results

Demography

Sex

Male preponderance was seen in bone tumors with 71.4% males (n=10) in benign bone tumors and 64.3% (n=9) in malignant bone tumors. Out of 14 controls, the male to female ratio was 2.5:1 i.e. 10 males and 4 females. The male-female ratio was comparable in all groups (p>0.05).

Age

The patient’s age varied from 6-62 years with a mean of about 22.5±12.6 years. Similarly, in the control group, age varied from 9-62 years with a mean of 21.3±10.8 years.

Location

Based on tumor location, the lower limb (n=19) was involved more commonly than the upper limb (n=9).

Side

Out of 28 lesions, 16 had left-sided lesions while 12 had lesions on the right side of the body.

Histopathological diagnosis of bone tumors

As seen in table 1, giant cell tumor (GCT) was the most common benign tumor (n=7) followed by aneurysmal bone cyst (ABC) (n=4). Among the remaining patients, two were diagnosed with enchondroma and one with chondroblastoma. On the other hand, osteosarcoma (n=9) was the most common malignancy seen on histopathology, followed by Ewing’s Sarcoma (n=4).

**Table 1:** Histopathological diagnosis of tumors

Benign tumors (n=14)	Malignant Tumors (n=14)
GCT (n=7)	Osteosarcoma (n=9)
ABC (n=4)	Ewings Sarcoma (n=4)
Enchondroma (n=2)	Chondrosarcoma (n=1
Chondroblastoma (n=1)	

Oxidative stress and antioxidant status

The levels of MDA and SOD in different groups are presented in Table 2 while a comparative analysis of p-values between different groups is shown in Table 3. The difference in the serum levels of both the parameters was found to be highly significant in malignant bone tumors as compared to healthy controls (p<0.001). Similarly, the association between malignant bone tumors and benign bone tumors was also found to be significant (p<0.05). It is pertinent to mention here that the difference between healthy controls and benign bone tumors was not found statistically significant (p>0.05).

**Table 2:** Comparison of MDA levels and SOD levels in different groups.

		Healthy controls (n=14)	Benign bone tumors (n=14)	Malignant bone tumors (n=14)
MDA (μmol/L)	Mean±S.D	2.36±1.10	5.72±3.2	7.40±4.10
	Range	1.02-3.54	3.24-8.52	4.85-9.33
SOD (IU/mL)	Mean±S.D	158±32	78±34	42±22
	Range	113-254	54-98	21-65

**Table 3:** p-value between different groups

	p-value between Healthy controls & benign bone tumors	p-value between Healthy controls & malignant bone tumors	p-value between benign bone tumors & malignant bone tumors
MDA (μmol/L)	0.1359	0.0005	0.0397
SOD (IU/mL)	0.0523	0.0075	0.0439

A negative non-significant correlation was found between MDA and SOD levels (r=-0.068, p=0.661) in malignant bone tumors, while the correlation in healthy controls (r=0.076, p=0.902) and benign bone tumors (r=0.210, p=0.430) was also not statistically significant. 

**Figure 1:**
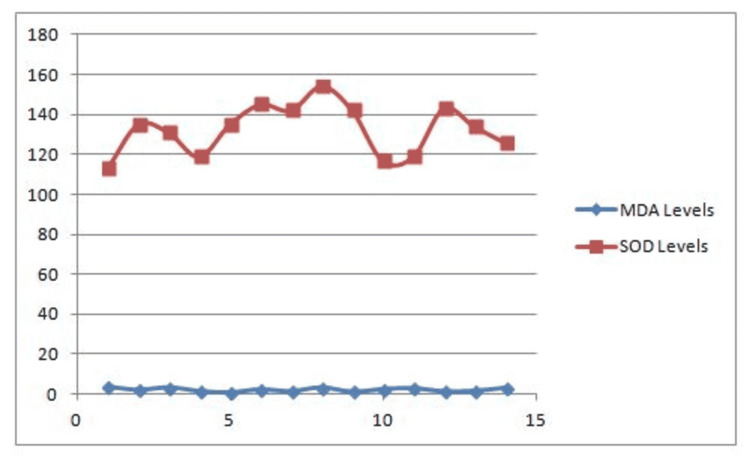
Correlation between MDA levels and SOD levels in Healthy Controls.

**Figure 2:**
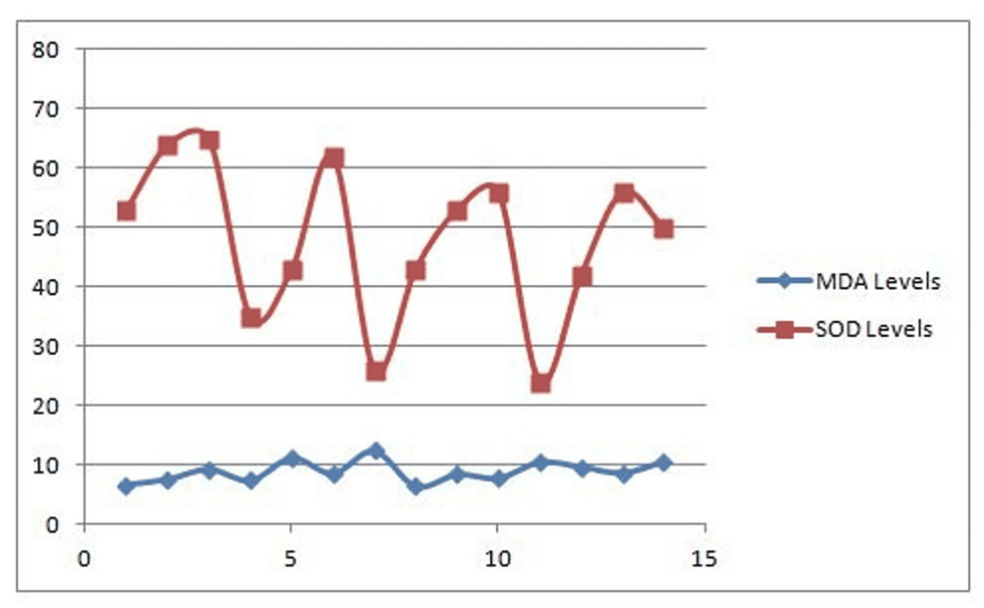
Correlation between MDA levels and SOD levels in Benign Bone tumors

**Figure 3:**
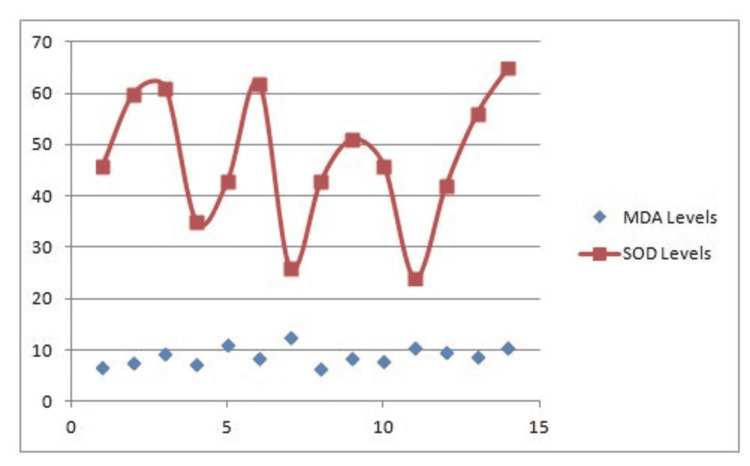
Correlation between MDA levels and SOD levels in Malignant bone tumors

Figures 1-3 are groupwise graphical representations of the results.

 

## Discussion

Many theories have been postulated to establish a correlation between oxidative stress and carcinogenesis. However, a concrete theory is yet to be established. This is a rare study that shows a significant increase in oxidative stress and a decrease in the antioxidant status in patients with benign as well as malignant bone tumors.

The active form of free radicals is involved in cell process regulation and indicates oxidative stress in a cell. The antioxidative system prevents the organism from the damage caused by oxidative stress. The imbalance between the oxidants-antioxidant systems has been investigated in many malignancies [9-11] including bony malignancies [2]. However, the levels of oxidative stress and antioxidant status in both benign, as well as malignant bone tumors are yet to be explored. Several studies have shown increased oxidative stress and decreased antioxidant status in various malignancies like breast, gastric, and cervical carcinoma as evident in Table 4.

**Table 4:** Levels of MDA and SOD in various malignancies as reported in literature.

Study (Year)	Type of Cancer	Serum MDA levels	Serum SOD levels
Nathan et al (2011)[2]	Bone & Soft tissue Sarcomas (n=47)	7.30 ± 4.10	34 ± 34
Sateesh et al (2019) [12]	Breast Cancer (n=30)	2.82 ± 0.73	-
Zahra et al (2020) [13]	Cervical Cancer (n=28)	3.77 ± 0.67	29.6 ± 6.2
Braga-Neto et al (2021)[14]	Gastric Cancer (n=50)	6.32 (3.94-12.13)	60 (48-73)
Present study	Bone tumors Benign (n=14) Malignant (n=14)	5.72±3.2 7.40±4.10	78±34 42±22

The measure of oxidative stress i.e. MDA levels were raised in both benign and malignant bone tumors as compared to healthy controls, though the rise in levels was more significant in malignant bone lesions. The above findings are consistent with the existing literature [12,13,14]. It is pertinent to mention here that though the levels were increased in both benign and malignant tumors, an increase in levels was not found statistically significant in benign bony lesions (p>0.05). The benign nature of lesions and less aggressive course of the disease could be one of the causes for the same as noted by Zahra et al where increased levels were seen with the increase in the staging of the disease [12]. Increased levels were also reported by Nathan et al in his study in patients with bone and soft tissue sarcomas which were statistically significant (p<0.05) [2].

Decreased antioxidant status as measured by SOD levels was observed in bone tumors, both benign and malignant, as compared to healthy controls. These results are consistent with the available literature. A possible explanation for the same could be due to the depletion of antioxidant enzymes to prevent damage to DNA, lipids, and proteins. Another possible theory is a weak defense mechanism of body leading to increased oxidation of DNA, lipids and proteins. Similar reductions have also been observed in breast cancer, cervical cancer, gastric cancer and many more.

The decrease in SOD levels was found to be statistically significant between healthy controls and malignant bone tumors with a p-value =0.000413, and between benign bone tumors and malignant bone tumors with a p-value =0.0407 which was quite significant. However, the difference between healthy controls and Benign bone tumors was not found to be significant (p>0.05). In his study, Nathan et al reported a significant decrease in levels of antioxidant enzymes in patients with bone and soft tissue sarcoma (p = 0.000) which are consistent with the findings of our study [2].

For early diagnosis and prevention, there is a need for new and effective markers, and there are many new markers coming up with time. Dahiya et al found 25-OH vitamin D and NGAL as effective biomarkers in patients with malignant bone tumors [15].

The small sample size remains one of the limitations of the study. Alteration in results due to differences in levels of cellular levels and serum levels of enzyme status remains a challenge. The establishment of oxidative stress as an aetiology of bone tumors can have both therapeutic as well as diagnostic implications.

## Conclusions

The present study measured oxidative stress (MDA levels) and antioxidant status (SOD levels) in benign as well as malignant tumors and found a statistically significant increase in levels of MDA and a decrease in SOD levels. These findings are consistent with findings of patients with colorectal cancer, gastric cancer, breast cancer. Although no association was found in benign bone tumors, statistically significant results were seen in malignant bone tumors. The same can be used for diagnostic as well as therapeutic purposes
